# An introduction to photon-counting detector CT (PCD CT) for radiologists

**DOI:** 10.1007/s11604-022-01350-6

**Published:** 2022-10-18

**Authors:** Yuko Nakamura, Toru Higaki, Shota Kondo, Ikuo Kawashita, Isao Takahashi, Kazuo Awai

**Affiliations:** 1 Diagnostic Radiology, Hiroshima University, 1-2-3 Kasumi, Minami-ku, Hiroshima, 734-8551 Japan; 2 Graduate School of Advanced Science and Engineering, Hiroshima University, 1-4-1 Kagamiyama, Higashi-Hiroshima, Hiroshima 739-8527 Japan; 3 FUJIFILM Healthcare Corporation, 2-1, Shintoyofuta, Kashiwa-shi, Chiba 277-0804 Japan; 4 FUJIFILM Corporation, 9-7-3 Akasaka, Minato-ku, Tokyo, 107-0052 Japan

**Keywords:** Photon-counting detector CT, Energy-integrating detector CT, Spatial resolution, Energy separation, K-edge imaging

## Abstract

The basic performance of photon-counting detector computed tomography (PCD CT) is superior to conventional CT (energy-integrating detector CT: EID CT) because its spatial- and contrast resolution of soft tissues is higher, and artifacts are reduced. Because the X-ray photon energy separation is better with PCD CT than conventional EID-based dual-energy CT, it has the potential to improve virtual monochromatic- and virtual non-contrast images, material decomposition including quantification of the iodine distribution, and K-edge imaging. Therefore, its clinical applicability may be increased. Although the image quality of PCD CT scans is superior to that of EID CT currently, further improvement may be possible. The introduction of iterative image reconstruction and reconstruction with deep convolutional neural networks will be useful.

## Introduction

Photon-counting detector CT (PCD CT) features a detector whose principle differs from that of conventional CT; it has been under technical development for more than ten years [1–6]. PCD CT can reduce the electronic circuit noise, the radiation dose, beam-hardening- and metal artifacts, increase the iodine contrast-to-noise ratio (CNR) and the spatial resolution, and improve X-ray energy separation [7, 8]. It is expected to be a new generation of X-ray CT, and the first clinical CT system to use PCD technology has become available for patient care (https://www.siemens-healthineers.com/en-us/press-room/press-releases/fdaclearacenaeotomalpa). Dual-energy CT (DECT) with conventional detectors has been clinically used for more than ten years. It was an advancement over single-energy CT because it increased the CNR of iodine, reduced the radiation dose, and improved the X-ray energy separation [9–12]. Not enough radiologists understand the difference between the principles of PCD CT and DECT and what advances in the CT diagnosis can be expected from PCD CT. Therefore, we present the principles of PCD CT to clinicians and discuss the potential clinical applications of PCD CT.

## Conventional versus photon-counting X-ray detector

### Energy-integrating detector for conventional CT

**Fig. 1:**
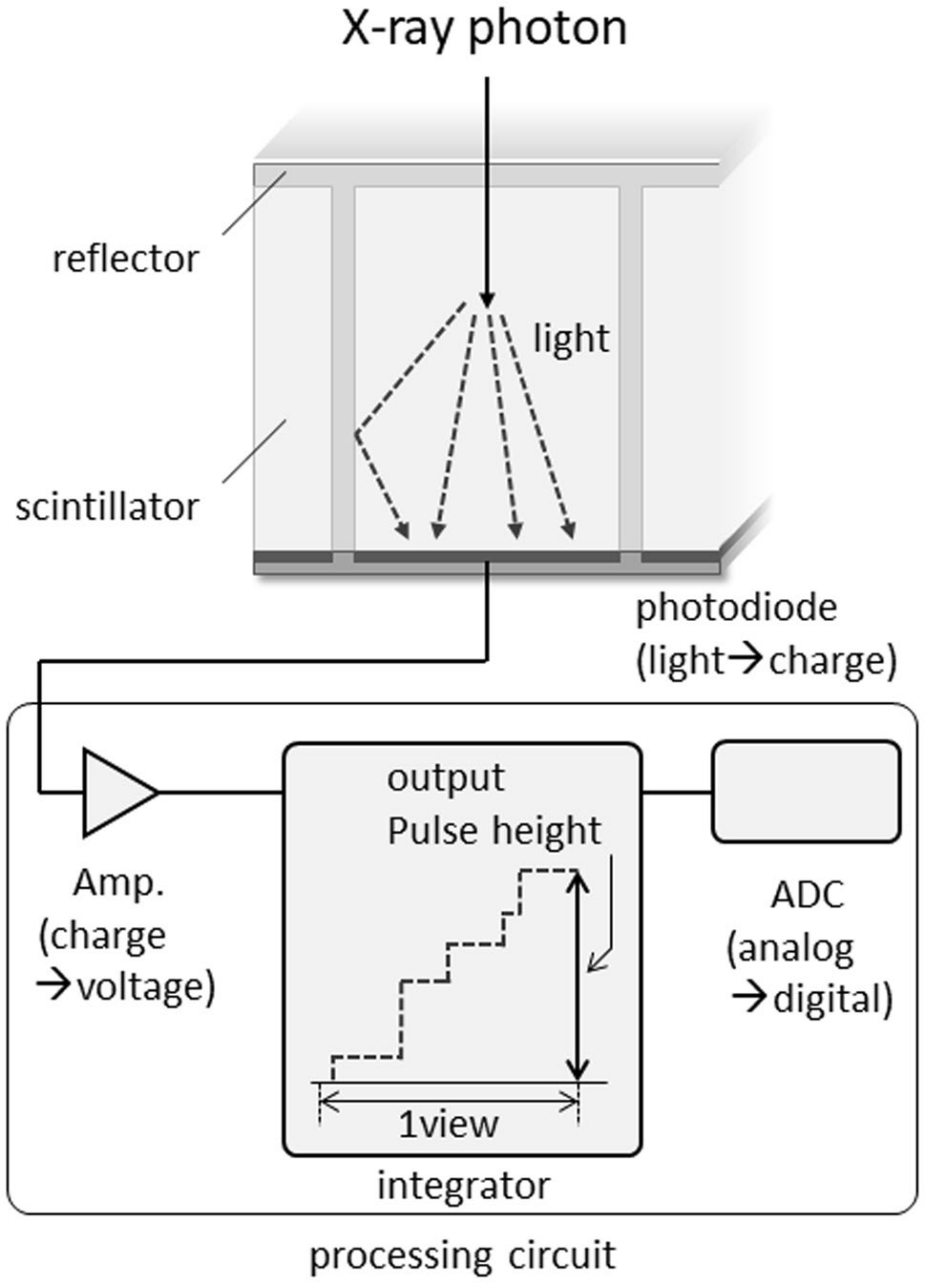
Schematic drawing of energy-integrating detector (EID) for conventional CT

Figure 1 is a diagram of the detector used in conventional CT studies. Most current CT detectors are solid-state scintillator detectors made of Gd_2_O_2_S or CdWO_4_. When X-ray photons enter the scintillator of a detector segment, scintillation light is generated by their interaction. After the scintillation light reaches the photodiode, the photodiode converts the scintillation light into an electrical signal. As each detector element is separated from the adjacent detector elements by separators, the generated scintillation light does not affect them. If the separators are thick, the efficiency of X-ray photon utilization in the plane is reduced, therefore, the separators must be thin. The spatial resolution on conventional CT scans depends on the width of the detector element, including the separator. At present, this width is 0.25–0.625 mm at the iso-center.

The generated scintillation light intensity is proportional to both the energy of each X-ray photon and the number of incident photons per unit time. As conventional CT detectors produce an electrical signal proportional to the total amount of scintillation light generated in the scintillator during the measurement time, they are also called energy-integrating detectors (EID). The electrical signal converted from the scintillation light by the photodiode is amplified and then integrated by the integrator and finally becomes the output signal.

### Photon-counting detector

**Fig. 2:**
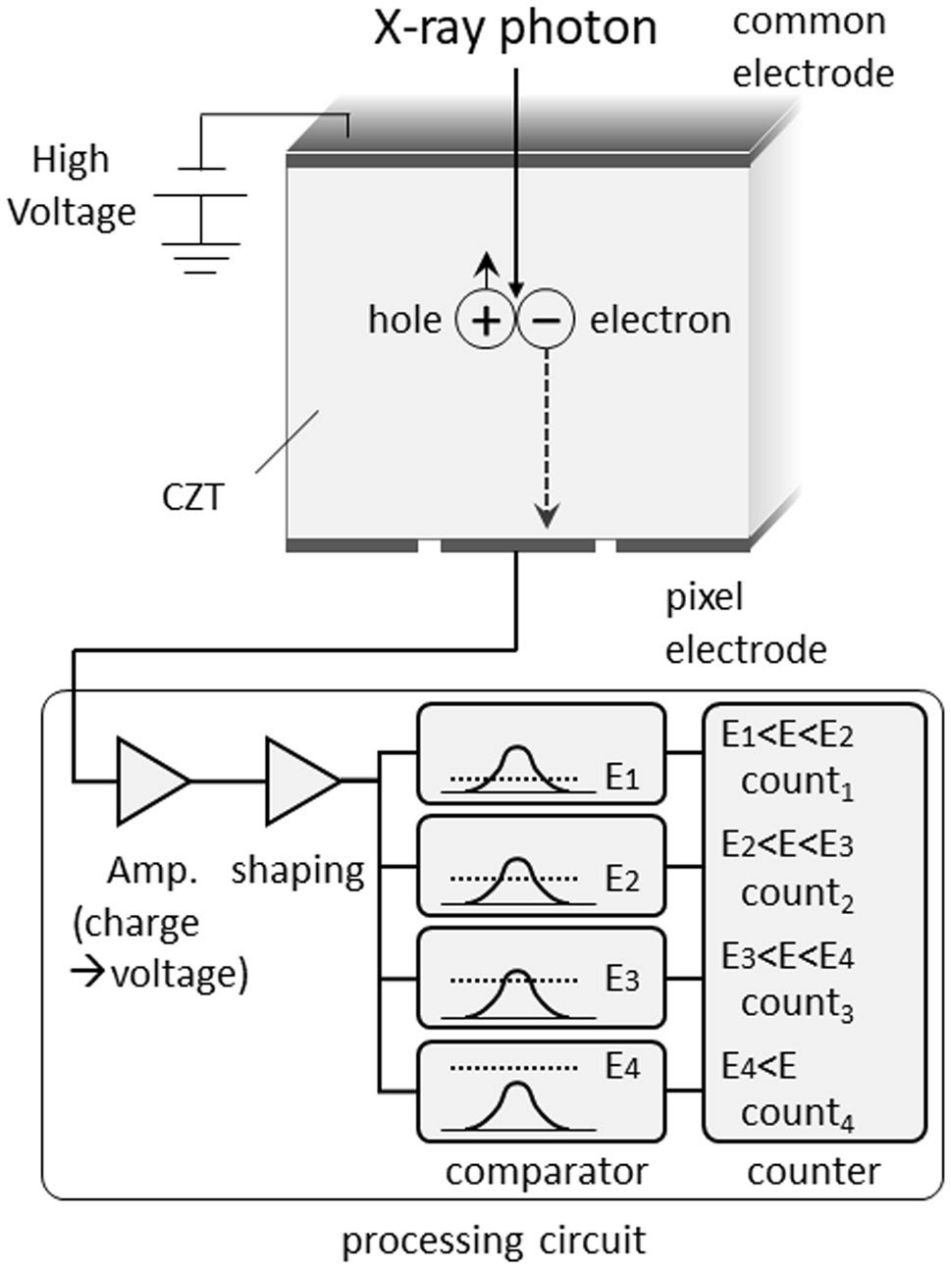
Schematic drawing of photon-counting detector (PCD)

Figure 2 is a diagram of the PCD. It is made of materials such as CdTe, CdZnTe, and silicon [1–3, 10, 13]. When the material is CdTe or CdZnTe, the probability of Compton scattering is low because their atomic number is large and their X-ray absorption efficiency is high. Consequently, highly accurate energy information can be obtained. On the other hand, in silicon, the probability of Compton scattering is higher. However, silicon is a mature semiconductor, and methods to obtain accurate energy information by data processing have been developed in silicon detectors [5].

**Fig. 3:**
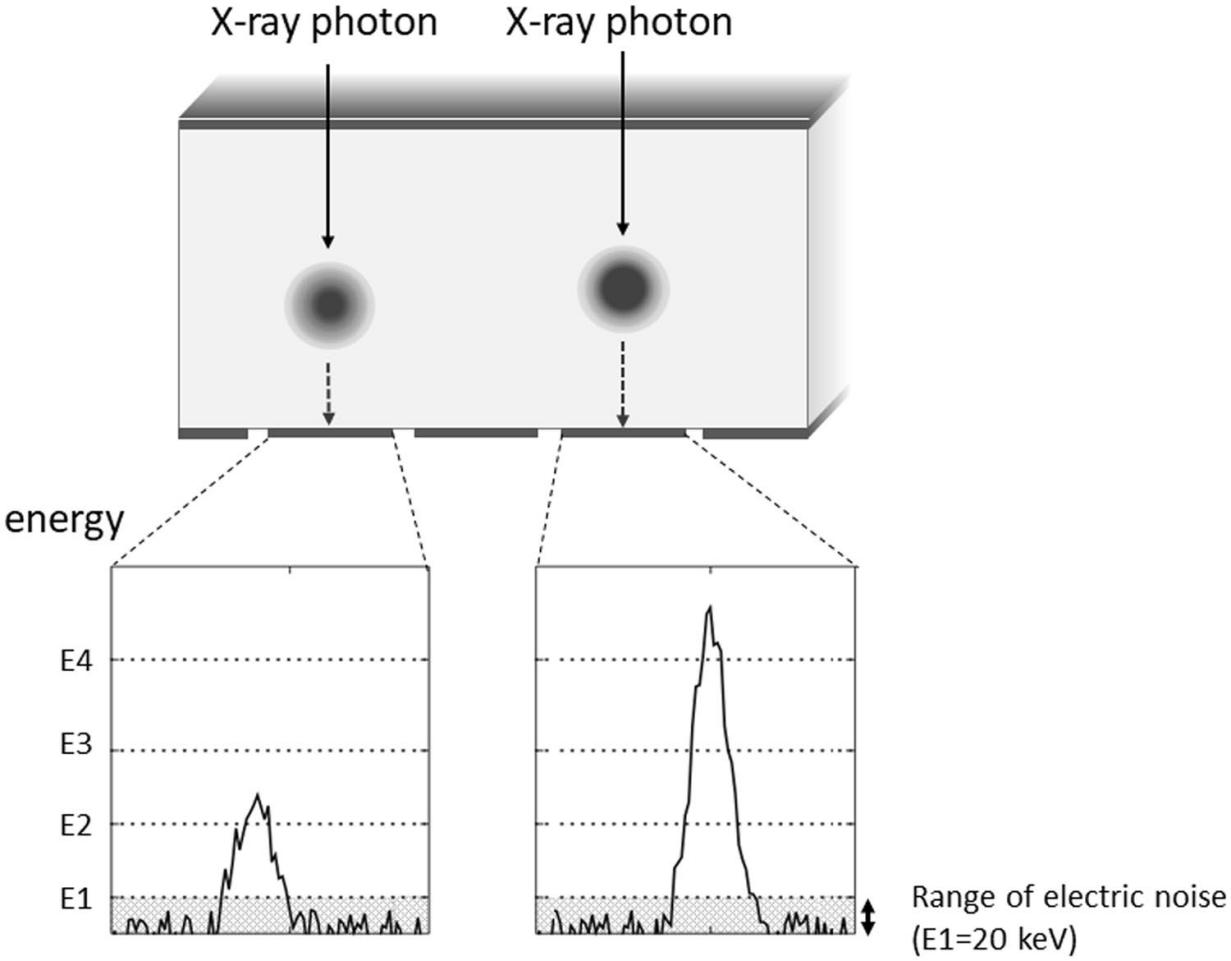
Incident X-ray photons into the detector and generation of pulse (ideal situation). Under ideal situations, one charge cloud caused by a single photon enters to one-pixel electrode and one pulse is generated. As pulse caused by electric noise is well lower than 20 keV, electric noise can be removed by setting the lowest energy threshold of PCD to around 20 keV

When X-ray photons enter the detector, their interaction with the detector generates a charge cloud of electron–hole pairs. The charge cloud can be swept to the pixel electrodes (anode electrode: anode pixel) by the electric field applied to the PCD, generating a pulse. Under ideal situations, one charge cloud generated by a single photon enters one pixel electrode perpendicularly and generates one pulse (Fig. 3). The spatial resolution on PCD CT scans depends on the size of the pixel electrode and a reduction in its size improves the spatial resolution. However, reducing the pixel electrode size increases the possibility of measurement errors due to charge-sharing and K-escape (see below). Unlike the EID, the PCD does not include separators and the concept of “multiple detector rows” cannot apply.

Electronic circuits that are connected to the anode and shape electric pulses and count the signals according to their energy values are called application-specific integrated circuits (ASICs) (Fig. 2). The charge cloud of electrons arriving at the pixel electrode generates a signal which is amplified and converted into voltage, and the voltage signal is shaped into a pulse by a “pulse shaper”. The shaped pulse is then sent to a “comparator”, which measures the energy value from the height of the pulse. Then, a “counter” measures how many pulses have a wave height that exceeds a preset energy threshold. For example, to obtain the number of pulses between the energy thresholds E1 and E2 (E1 < E2), the number of pulses higher than E1 is subtracted from E2 and the PCD classifies incident photons into several energy bins based on their energy by comparing all pulses with several thresholds. The energy bins of current PCD CT range from 2 to 8.

As PCD CT reduces the electronic noise in imaging, the image quality of low-dose-scans and scans of patients with a large body size is improved. There are two types of noise on CT images, i.e., quantum noise and electronic noise. Quantum noise originates from the fluctuation in the number of incident photons on the X-ray detector and depends on the incident radiation dose delivered to the detector. Electronic noise is produced by the electronic circuit of the detector. When the radiation dose is high, the electronic noise can be largely ignored because the quantum noise accounts for most of the total noise. However, it cannot be ignored when the radiation dose is low. Electronic noise is usually observed as pulses with an energy lower than 20 keV. Therefore, if the lowest energy threshold in the energy counter of the PCD CT scan is set to around 20 keV, the electronic noise can be removed efficiently (Fig. 3).

The contrast is good on PCD CT scans because the number of all photons from low- to high energy is equally counted. At EID CT, on the other hand, low-energy photons contribute less to the output signal and contrast is lower. PCD CT can improve the contrast of soft tissues as the energy information on human organs is distributed in the relatively low-energy X-ray spectrum.

### Physical issues of the photon-counting detector CT

#### Cross talk

**Fig. 4:**
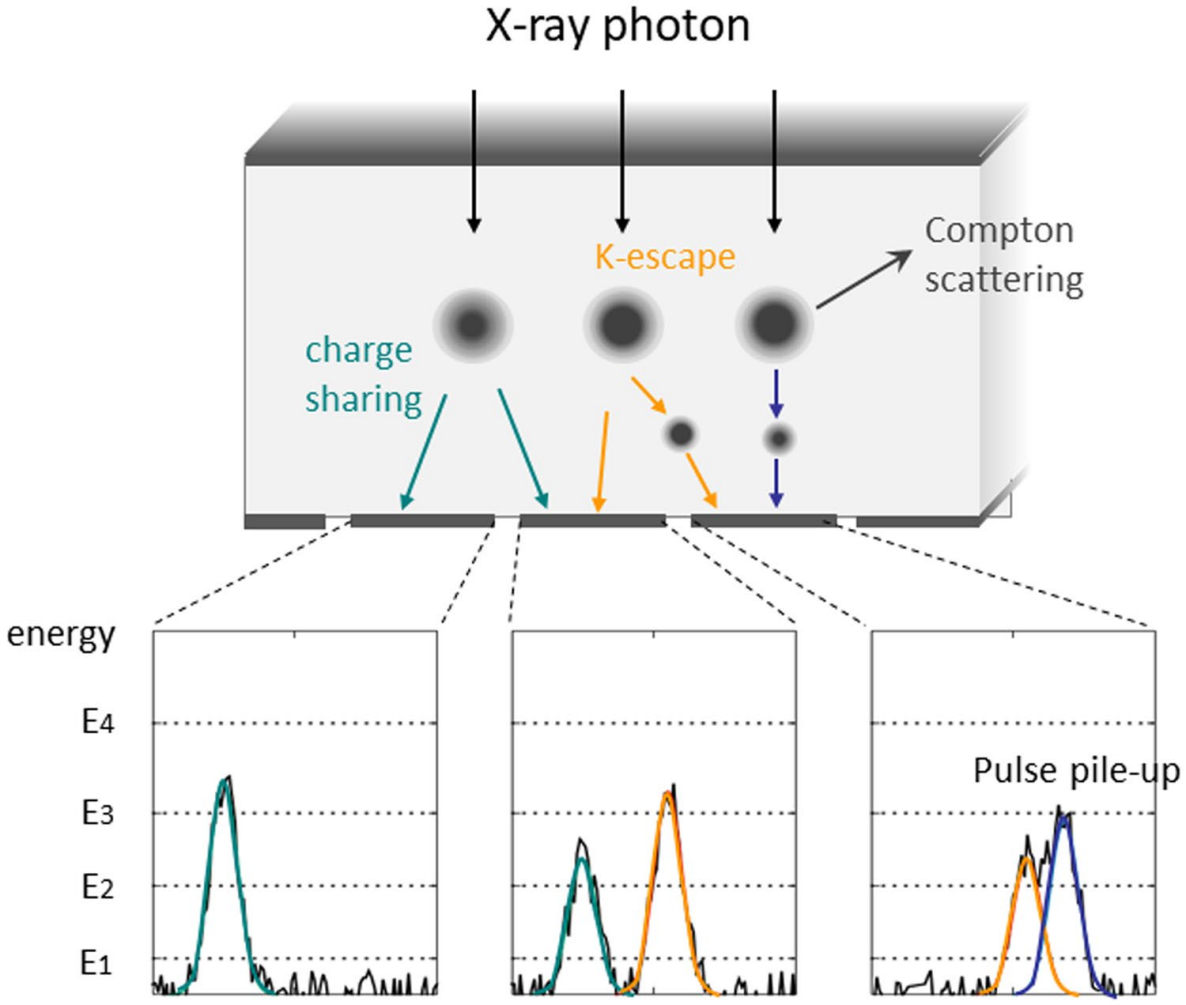
Charge sharing, K-escape, and Compton scattering causing inaccurate signal measurements in the PCD

Ideally, the charge cloud arising in the PCD is swept to a single pixel electrode and a pulse is generated (Fig. 3). Actually, however, charge-sharing, K-escape, and Compton scattering can take place in the PCD and inaccurate signal measurements are obtained (Fig. 4).

Charge-sharing occurs when a charge cloud is near the boundary of a pixel electrode and the cloud is counted in multiple adjacent pixel electrodes. K-escape is seen when a new charge cloud is generated by the Kα X-ray fluorescence of the sensor materials (Cd and Te) in addition to the original charge cloud. X-ray photons sometimes interact with the detector material by Compton scattering, and only a small fraction of their energy may be deposited at the detector element. Also, the direction of scattered photons is not predictable and the remaining energy may reach another detector element. The effects of Compton scattering are more significant with silicon detectors [7].

**Fig. 5:**
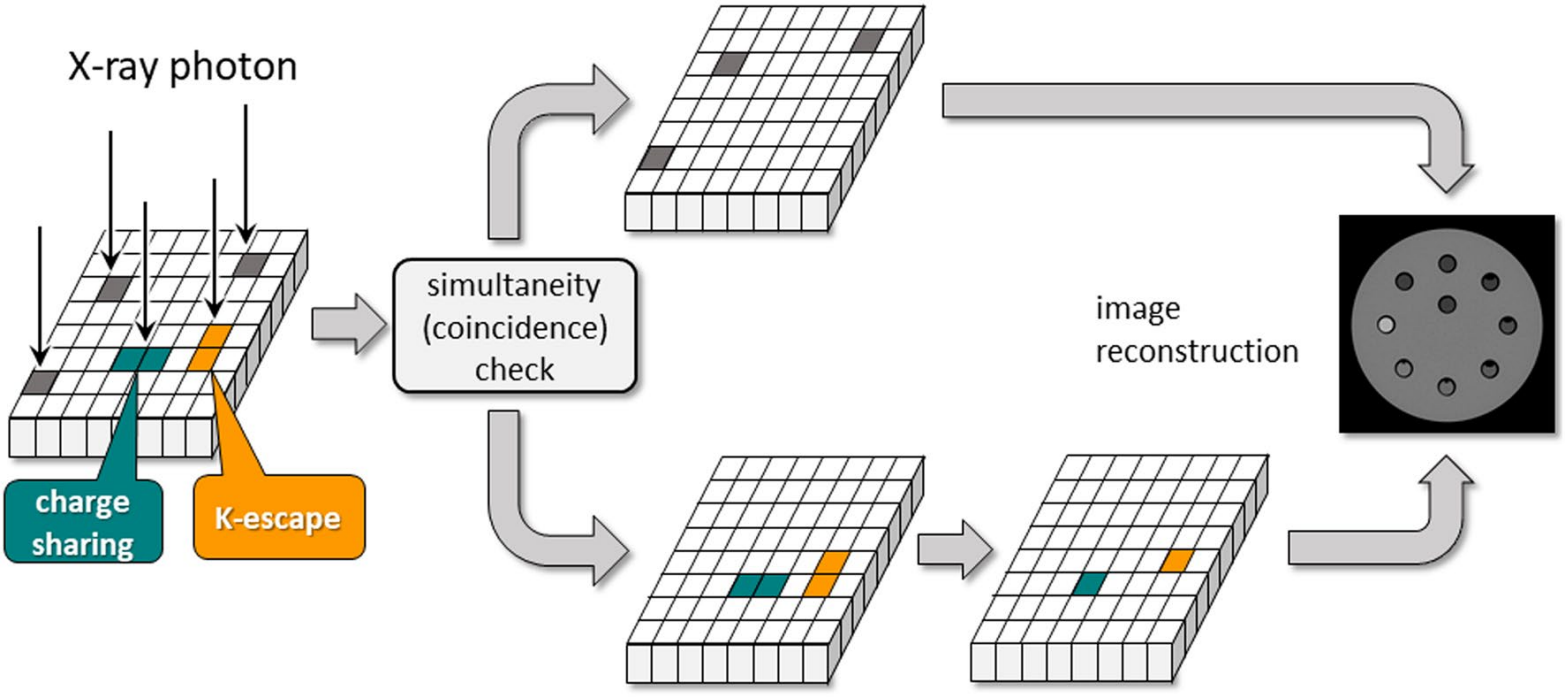
A method to correct split signals. To handle split signals caused by, e.g., charge sharing or K-escape, simultaneity of detected signals is checked in the pixel electrodes

A check of the simultaneity (coincidence) of detected signals in the pixel electrodes helps to manage charge-sharing and K-escape. This method can also handle Compton scattering when the scattered photon is absorbed and detected in the adjacent pixel electrode; each detected signal is checked to determine whether other signals in adjacent pixels are detected simultaneously or within a short time window. To correctly reflect the actual number of incident photons, when two or more signals are simultaneously detected, they are converted into a single signal by synthesizing the signals which can be generated by charge-sharing or K-escape on the basis of the detected energy. The original signal is identified based on the pulse height (energy) of the detected simultaneous signals. Thus, the simultaneous signals are corrected to yield actual information on the incident photons and they are added to non-simultaneous signals and used for image reconstruction (Fig. 5).

#### Pulse pile-up

In CT, several hundreds of millions of photons per square millimeter per second are incident on the detector [5, 14]. The detector material must rapidly transport the generated charge cloud to the pixel electrode and the ASICs must count the generated pulses very quickly. Therefore, the detectors and ASICs must be capable of processing a photon within tens of nano-seconds. Because the newly developed detectors and ASICs process pulses at high speed, PCD CT can now be used clinically.

When the signal processing of the detector is relatively slow, “pulse pile-up” occurs and some of the generated electric pulses overlap. Two consecutive, almost simultaneous pulses are registered as a single pulse and the count is underestimated. Although the detector registers pulses with a slight difference in their arrival time as two separate counts, their partial overlap results in an error in the measured photon energy. When the superposition of the pulses identifies the pulse height as larger than it should be, the energy value is overestimated.

**Fig. 6:**
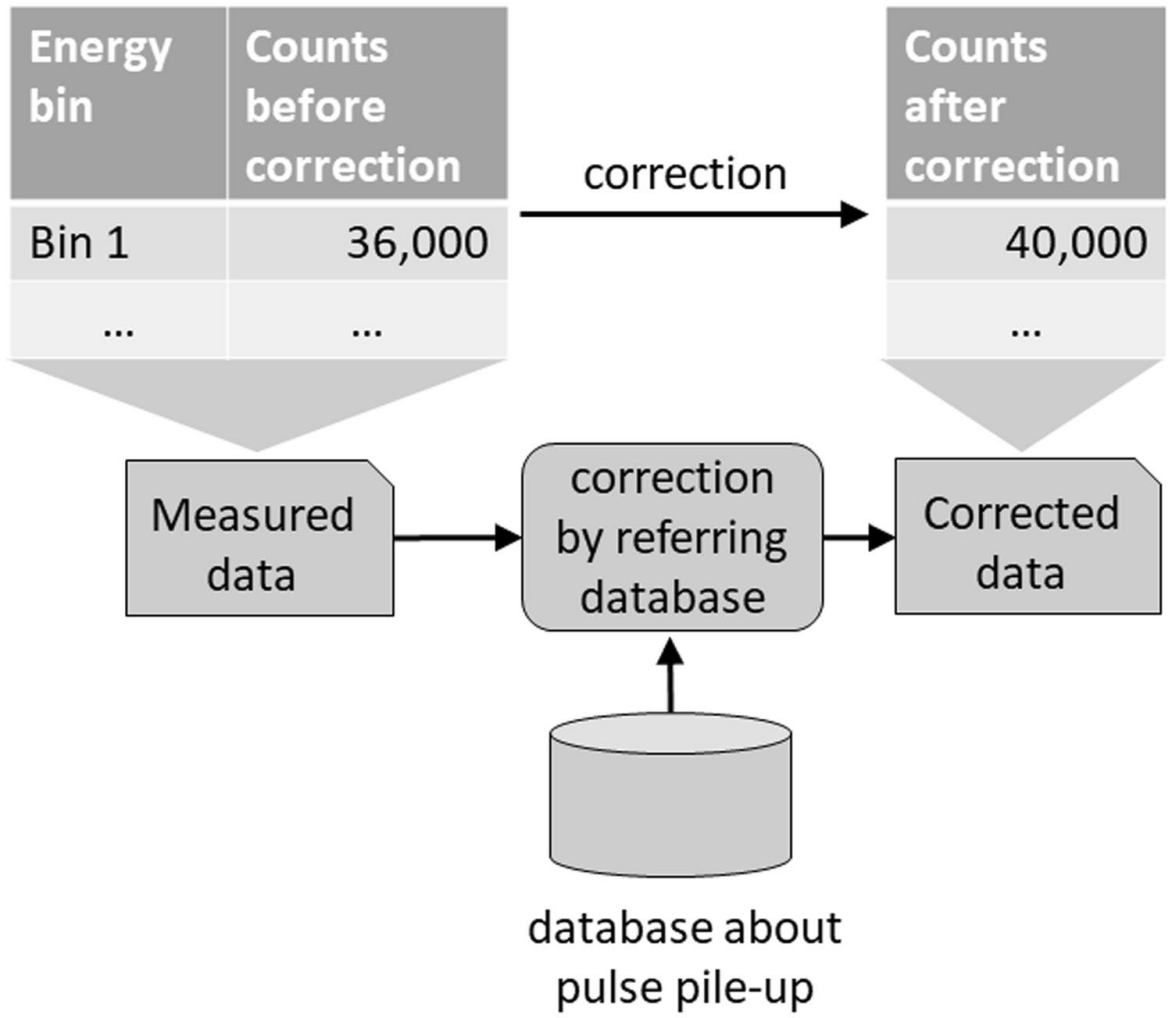
A method to correct pulse pile-up. A method to correct data affected by pulse pile-up is referring to a previously prepared database of signal deterioration due to pulse pile-up

By making the detector pixels smaller, the number of incident photons per detector channel can be reduced, and the probability of pulse pile-up is decreased. However, when the detector pixels are too small, the probability of charge-sharing increases and signals are generated separately in adjacent pixels. Although pulse pile-up can be corrected, it is best avoided; consequently, the pixel size must be optimal. Data affected by pulse pile-up can be corrected by referring to a prepared database of signal deterioration due to pulse pile-up (Fig. 6). This method can simultaneously correct pulse pile-up and yield accurate information on material decomposition.

### Material decomposition in the PCD CT

Material decomposition in DECT estimates the density of two basis materials by using the linear attenuation coefficients of materials scanned at two X-ray energies as:

1 $$\begin{gathered} \mu \left( {E_{1} } \right) = c_{{\text{A}}} \mu_{{\text{A}}} \left( {E_{1} } \right) + c_{{\text{B}}} \mu_{{\text{B}}} \left( {E_{1} } \right) \hfill \\ \mu \left( {E_{2} } \right) = c_{{\text{A}}} \mu_{{\text{A}}} \left( {E_{2} } \right) + c_{{\text{B}}} \mu_{{\text{B}}} \left( {E_{2} } \right) \hfill \\ \end{gathered}$$

where *μ*_A_(*E*_*x*_) and *μ*_B_(*E*_*x*_) represent the experimentally known linear attenuation coefficients of materials A and B at energy *E*_*x*_, and *c*_A_ and *c*_B_ the density of unknown basis materials A and B. Equation 1 is a binary linear system, and the density of basis materials can be estimated by finding the solution.

Current PCD CT allows scanning in 2–8 energy bins. If the object is scanned in 2-bin mode, the method in DECT can be applied as is. If the object is scanned in 3 or more energy bins, there are two possible analysis options (see Eqs. 2 and 3). For example, if the object is scanned at four energy bins, Eq. 2 estimates the two unknowns (density of the basis materials) from the four simultaneous equations. This method is almost the same as the DECT method. Although it can only estimate the density of two basis materials, since the number of equations is larger than the number of unknowns, it provides a robust solution that is less susceptible to noise.

2 $$\begin{gathered} \mu \left( {E_{1} } \right) = c_{{\text{A}}} \mu_{{\text{A}}} \left( {E_{1} } \right) + c_{{\text{B}}} \mu_{{\text{B}}} \left( {E_{1} } \right) \hfill \\ \mu \left( {E_{2} } \right) = c_{{\text{A}}} \mu_{{\text{A}}} \left( {E_{2} } \right) + c_{{\text{B}}} \mu_{{\text{B}}} \left( {E_{2} } \right) \hfill \\ \mu \left( {E_{3} } \right) = c_{{\text{A}}} \mu_{{\text{A}}} \left( {E_{3} } \right) + c_{{\text{B}}} \mu_{{\text{B}}} \left( {E_{3} } \right) \hfill \\ \mu \left( {E_{4} } \right) = c_{{\text{A}}} \mu_{{\text{A}}} \left( {E_{4} } \right) + c_{{\text{B}}} \mu_{{\text{B}}} \left( {E_{4} } \right) \hfill \\ \end{gathered}$$

The other option uses Eq. 3. It estimates the four unknowns (*c*_A_ to *c*_D_: the density of basis materials A to D) from the four simultaneous equations. This method may provide more accurate material decomposition than the method using Eq. 2. However, due to the reduced robustness of the solution and insufficient evidence for the analysis assuming four basis materials, analysis using Eq. 2 is generally used.

3 $$\begin{gathered} \mu \left( {E_{1} } \right) = c_{{\text{A}}} \mu_{{\text{A}}} \left( {E_{1} } \right) + c_{{\text{B}}} \mu_{{\text{B}}} \left( {E_{1} } \right) + c_{{\text{C}}} \mu_{{\text{C}}} \left( {E_{1} } \right) + c_{{\text{D}}} \mu_{{\text{D}}} \left( {E_{1} } \right) \hfill \\ \mu \left( {E_{2} } \right) = c_{{\text{A}}} \mu_{{\text{A}}} \left( {E_{2} } \right) + c_{{\text{B}}} \mu_{{\text{B}}} \left( {E_{2} } \right) + c_{{\text{C}}} \mu_{{\text{C}}} \left( {E_{2} } \right) + c_{{\text{D}}} \mu_{{\text{D}}} \left( {E_{2} } \right) \hfill \\ \mu \left( {E_{3} } \right) = c_{{\text{A}}} \mu_{{\text{A}}} \left( {E_{3} } \right) + c_{{\text{B}}} \mu_{{\text{B}}} \left( {E_{3} } \right) + c_{{\text{C}}} \mu_{{\text{C}}} \left( {E_{3} } \right) + c_{{\text{D}}} \mu_{{\text{D}}} \left( {E_{3} } \right) \hfill \\ \mu \left( {E_{4} } \right) = c_{{\text{A}}} \mu_{{\text{A}}} \left( {E_{4} } \right) + c_{{\text{B}}} \mu_{{\text{B}}} \left( {E_{4} } \right) + c_{{\text{C}}} \mu_{{\text{C}}} \left( {E_{4} } \right) + c_{{\text{D}}} \mu_{{\text{D}}} \left( {E_{4} } \right) \hfill \\ \end{gathered}$$

**Fig. 7:**
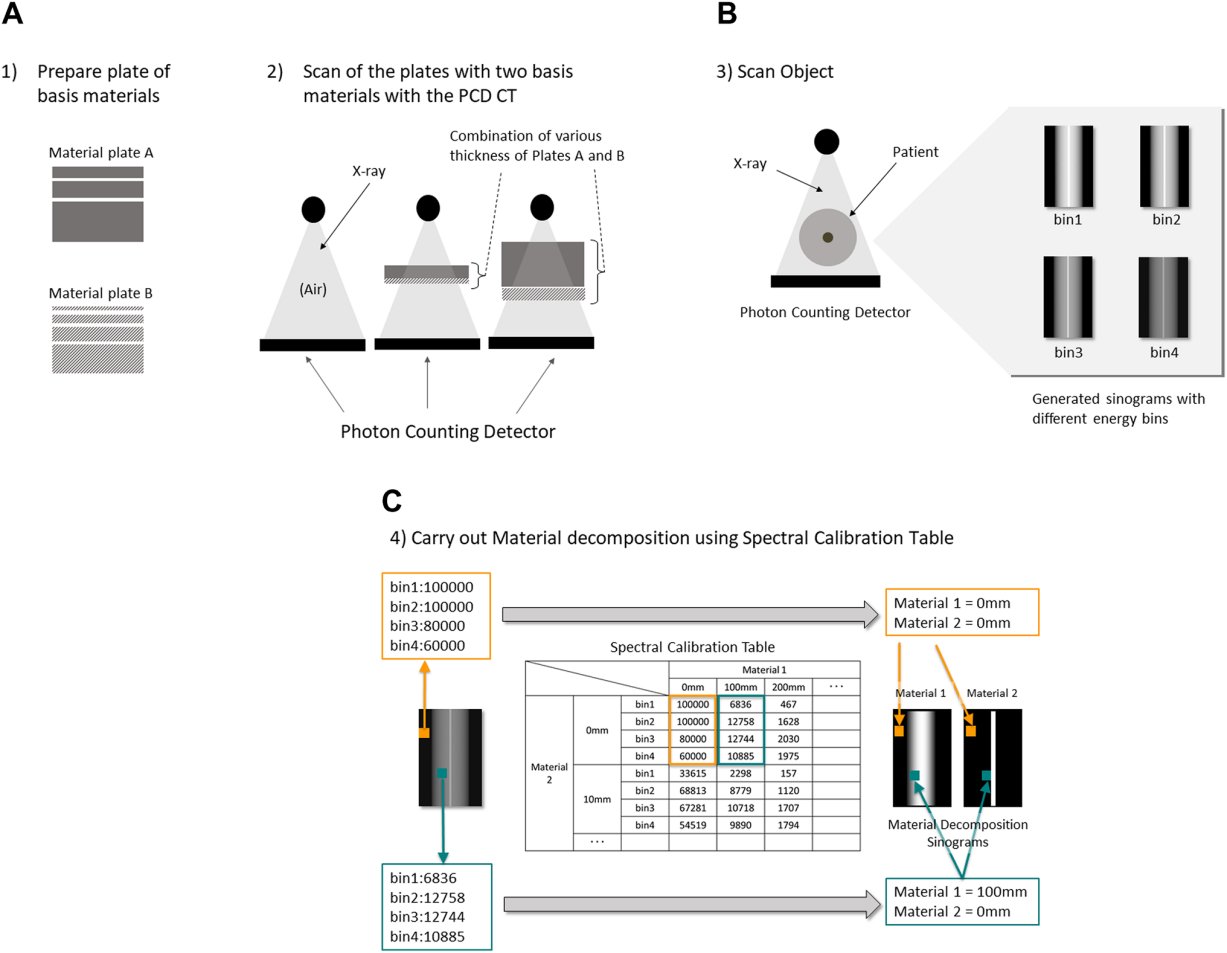
A method for material decomposition in the PCD CT. This method uses a previously prepared reference table (the values in figure are not actual measurements, but only show concepts)

What follows is a practical method for material decomposition in PCD CT (Fig. 7). First, prepare plates of various thicknesses whose X-ray absorption is equal to that of the basis materials (e.g., water, calcium). Select any two basis materials and combine basis material plates with different thicknesses. For example, if, in addition to a plate with a thickness of 0, there are 3 basis material A plates with different thickness (> 0 mm) and 4 basis material B plates, there will be 20 [4(1 + 3) × 5(1 + 4)] combinations of basis materials A and B. For all these combinations of the plates, the scans are carried out to prepare spectral calibration table beforehand, which contains counts for each energy bin of each pixel for various thickness of basis materials. Next, scan the object with PCD CT and obtain the count number in each pixel on the sinogram of each energy bin. Determine the thickness of the basis material contained in the X-ray path of each pixel by comparing the count in each pixel on the sinogram of each energy bin with the spectral calibration table and generate two sinograms representing the thickness of the two basis materials. Finally, reconstruct axial images of the two basis materials from the sinograms of the two basis materials. Then generate a virtual monochromatic image in the same way as with dual-energy CT. According to Alvarez and Macovski [15], the X-ray attenuation properties of the human body can be explained by the combination of two basis materials. Therefore, this method assumes that the human body consists of a combination of only two basis materials. The advantage of this method is that it can generate better quality of the images using the energy information since the nonlinearity of the detector can be corrected with high accuracy. The disadvantage of this method is that only the materials in the spectral calibration table can be assumed as basis materials. However, new methods to freely determine the basis materials is under investigation [16, 17].

For all three equations described above, we assume a situation where there is no noise or statistical fluctuations. It is difficult to reduce statistical fluctuations in particular. High energy bins may have greater fluctuations because the number of photon is lower in the higher compared with lower energy region. Therefore, these fluctuations may result in the inaccurate CT number, degradation of image quality due to increase in artifact and decrease in signal-to-noise ratio, and also in the inaccurate result of analysis. If the width of the threshold level in the high energy region is set wider so that the number of photons increases, the fluctuations may be minimized. In addition, as each energy bin has a range of energy, it is difficult to approximate the data acquired in each energy bin with a single energy perfectly. Beam hardening effect is not considered in the equations although it may cause the inaccurate evaluation of energy of photon. Thus, the analysis method must be improved to eliminate the effect of these factors in material decomposition.

### Prototype clinical PCD CT scanner

What follows is an overview of PCD CT on a prototype scanner developed by FUJIFILM Healthcare Corporation (the instrument is currently not approved for clinical use). The specifications of the prototype PCD CT scanner are presented in Table 1. The Z-coverage is 10 mm at the iso-center. This instrument was developed using a commercially available 64 detector-row EID CT scanner (SCENARIA View (system software V1.09), FUJIFILM Healthcare Corporation., Kashiwa, Japan); the detector was replaced with a photon-counting detector. The prototype features multi energy discrimination (MED)- and ultra-high resolution (UHR) detector mode.

**Table 1:** Specifications of the prototype PCD CT scanner

Specification		
Platform	SCENARIA view (system software version V1.09)	
Field of view	Max. 426 mm in-plane	
Gantry rotation	0.35, 0.5, 1.0 s	
View rate	2880 view/sec	
Tube voltage	120 kVp	
Tube current	Max. 300 mA	
Focal spot	0.7 × 0.8 mm, 1.2 × 1.4 mm	
Detector	MED mode	UHR mode
Energy bins	4*	1
Z-cover in iso-center	10 mm	
Detector pixel pitch in iso-center	0.58 × 0.63 mm	0.19 × 0.21 mm

*The threshold level for each energy bin is 30, 45, 65, and 90 keV

#### Ultra-high resolution (UHR) mode

This mode focuses on the utilization of the finer spatial information. The detected counts of all energy bins are added and processed. One spatially finer sinogram than the conventional CT sinogram is obtained. The main features of the UHR mode are that it can generate images with high spatial resolution using the finest unit of detector data and that it does not utilize X-ray energy information. The detector pixel pitch in UHR mode is 0.19 × 0.21 mm at the iso-center. The contrast differs from EID CT because the weighting factors for photons with various energies in the PCD CT are different from the EID CT as described before. That is, every photon equally results in 1 count in this mode, while higher energy photons create more signals in the energy-integrating system.

**Fig. 8:**
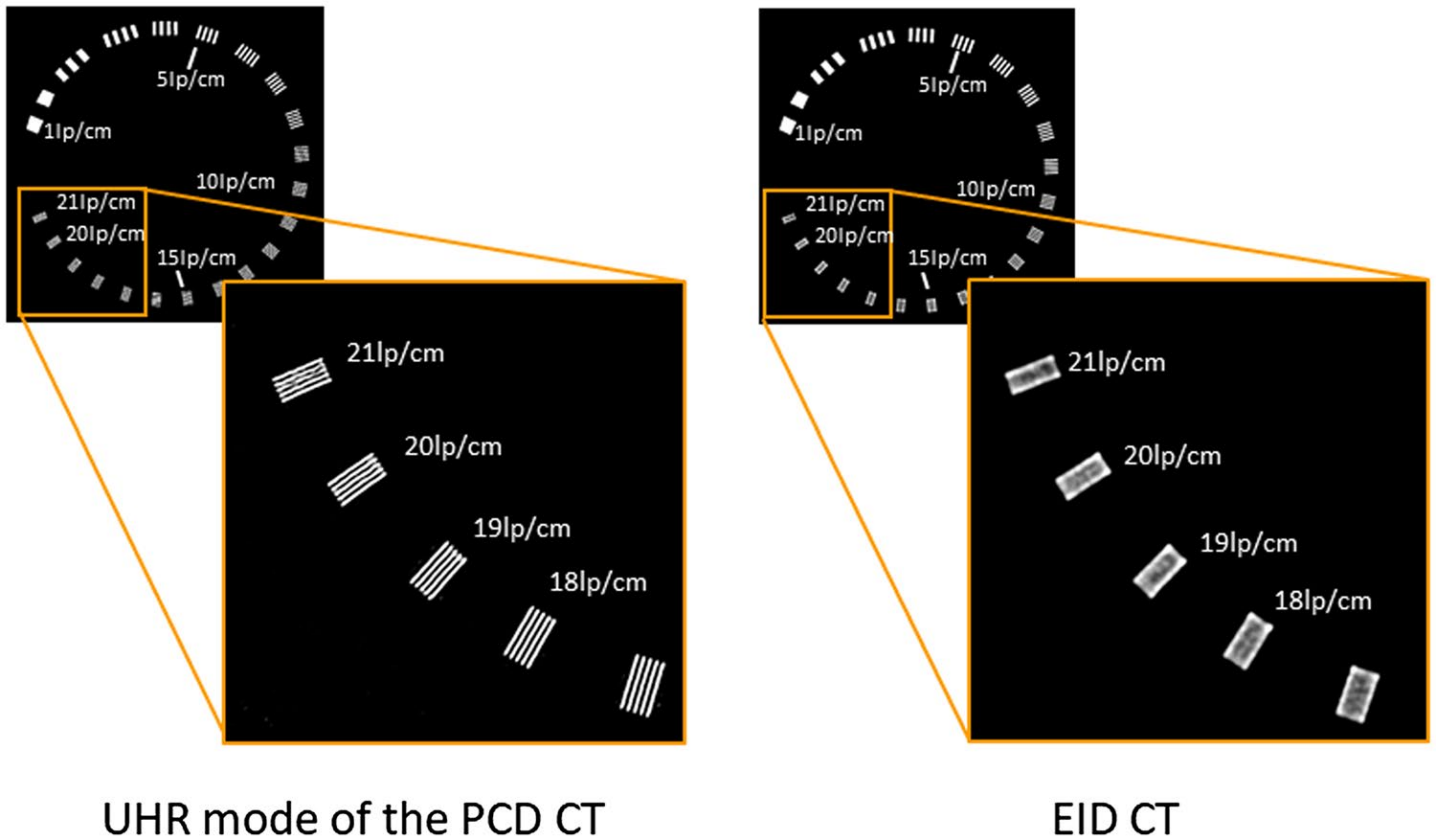
Comparison of spatial resolution in the phantom images between UHR mode of the PCD CT and EID CT (unpublished our own data). The phantom used for this imaging is Catphan 500 with CTP528 High Resolution Module (Phantom Laboratory Inc., Greenwich, USA)

**Fig. 9:**
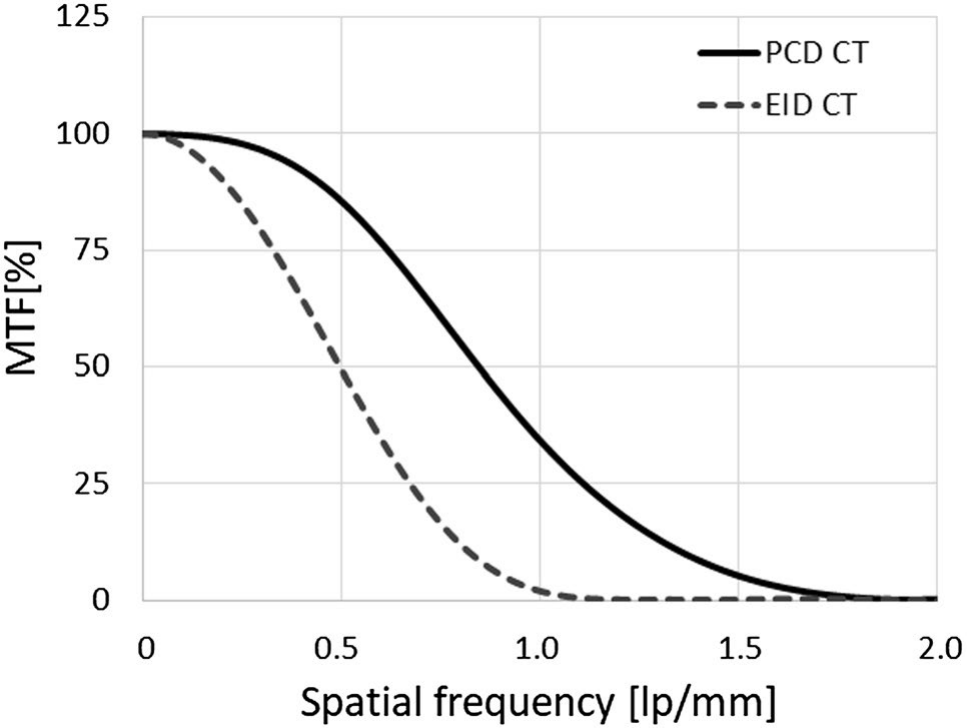
Comparison of modulation transfer function (MTF) between PCD CT and EID CT. The MTF for PCD CT shows higher response than that for EID CT in all frequency domains (unpublished our own data)

Figure 8 compares phantom images obtained with the UHR mode of the PCD CT and EID CT. The convolution kernel with the highest resolution in EID CT was used for both PCD CT- and EID CT. Although the UHR mode can separately delineate slits with 17–21 lp/cm, the EID CT cannot. The spatial resolution in the conventional EID CT is almost comparable to in MED mode of the PCD CT. Modulation transfer function (MTF) for PCD CT and EID CT was measured using metal wire technique. For MTF measurement the convolution kernel for soft tissue with the highest resolution was used for EID CT. The different convolution kernel which suppresses high-frequency components was used for PCD CT because of noise increment and appearance of Moiré-like artifact due to small element size. Figure 9 shows that the MTF for PCD CT shows higher response than that for EID CT in all frequency domains although the convolution kernel used for PCD CT yields lower response in MTF compared with the convolution kernel used for EID CT. These data were unpublished our own data.

#### Multi energy discrimination (MED) mode

**Fig. 10:**
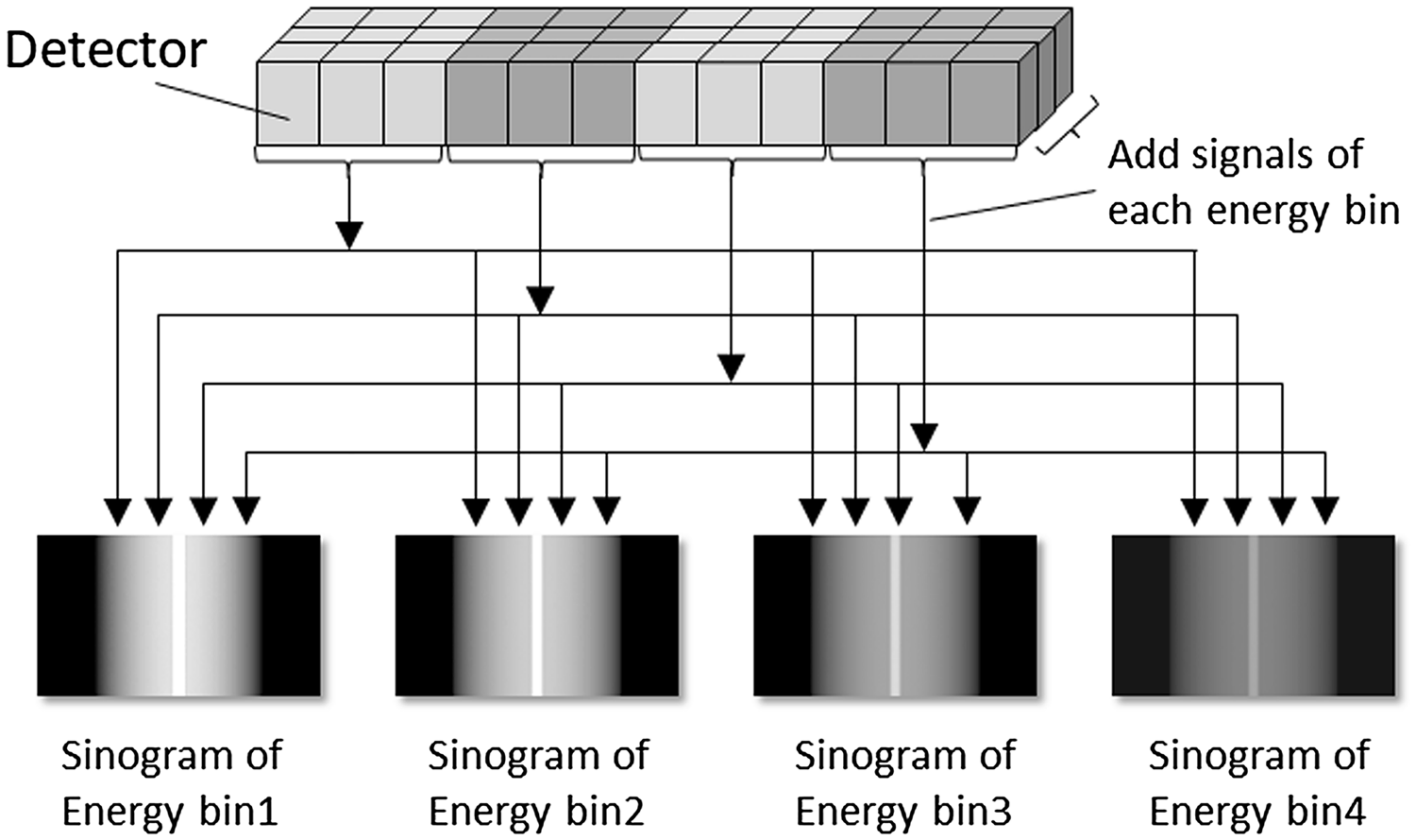
Schematic drawing of multiple energy discrimination (MED) mode of the PCD CT. In the MED mode, nine pixel electrodes are bundled together and treated like a single detector segment and the counts of the nine pixel electrodes are added for each energy bin (unpublished our own data)

In MED mode, nine (3 × 3) pixel electrodes are bundled together and treated like a single detector segment (Fig. 10). This clustered pixel electrode is called a detector macro-pixel and its size is almost the same as the detector segment of conventional EID CT. The spatial resolution in MED mode (0.58 × 0.63 mm at the iso-center) is almost equal to conventional EID CT; for each energy bin, the counts of the 9 pixel electrodes are added. For example, if data acquisition is performed in four energy bins, four sinograms with different energy bins will be generated.

**Fig. 11:**
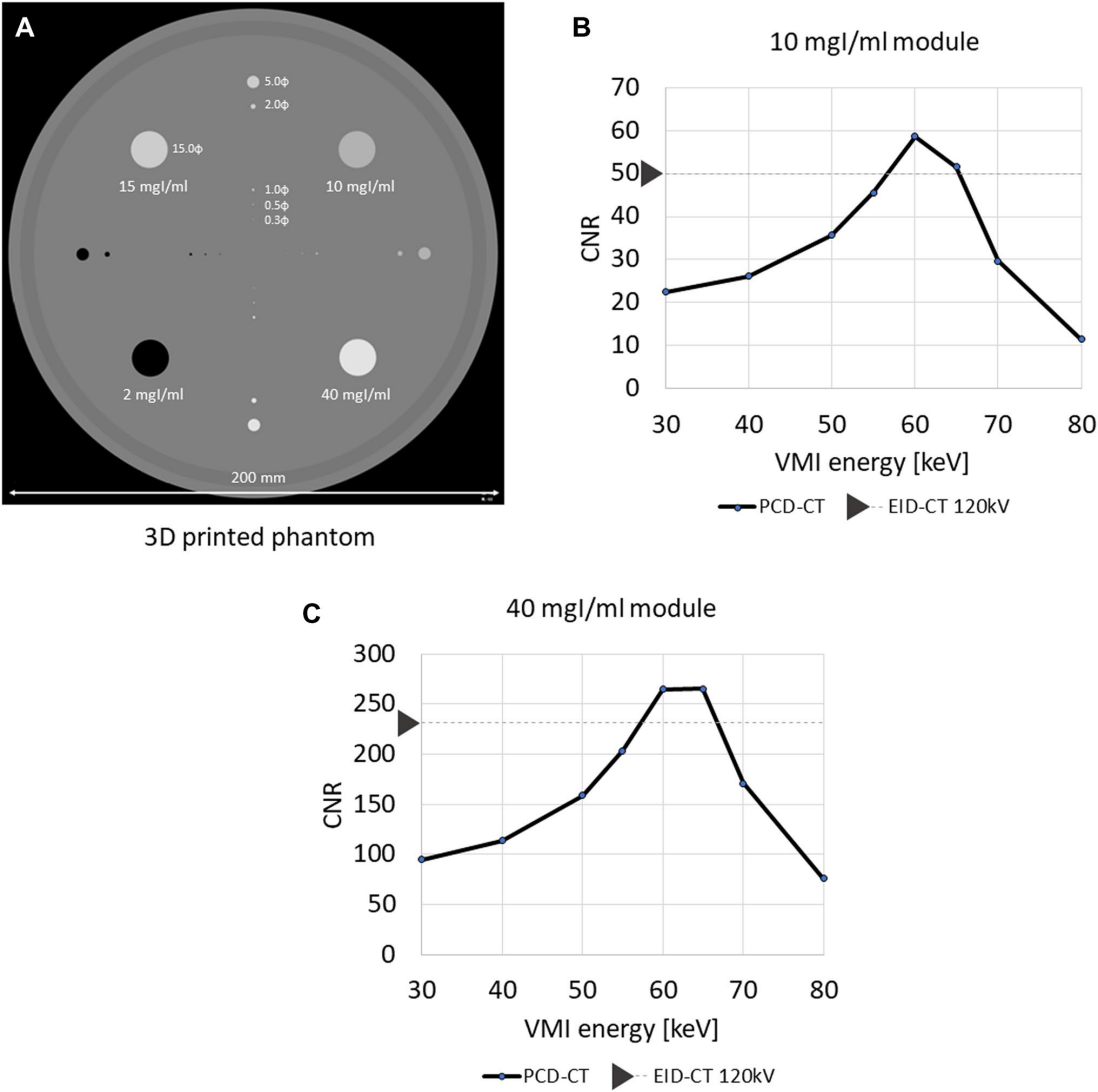
Iodine contrast-to-noise ratio (CNR) for each effective energy of virtual monochromatic images (VMI) generated from the PCD CT (unpublished our own data). **A** Configuration of the phantom to measure iodine CNR. The phantom is made of acrylate plastic and includes modules with different iodine concentration solutions. **B** and **C** Iodine CNR on the VMI generated from the PCD CT. Graphs show iodine CNR on the vertical axis and the effective energy (keV) of the VMI generated from the PCD CT on the horizontal axis. Iodine CNR on EID CT image is also shown on the vertical axis in each graph for reference

MED mode uses energy information to produce virtual monochromatic-, material-resolved-, virtual non-contrast-, virtual non-calcium-, effective atomic number-, and electron-density images. To measure the CNR of iodine, we scanned a phantom featuring a module containing iodine solution with PCD CT and EID CT (tube voltage 120 kV). For PCD CT scanning with MED mode the threshold level for each energy bin was set at 30, 45, 65, and 90 keV. As the number of photon is lower in the higher compared with lower energy region, the width of the threshold level in the high-energy region was set wider. In addition, because lower energy photons are considered to have more contrast information than higher energy photons, the width of the threshold level in the lower-energy region was set narrower. The phantom was made on a 3D printer; the body was of acrylate plastic and the modules contained different concentrations of an iodine solution (Fig. 11A). Figure 11B and C shows the CNR of iodine on the vertical axis; the effective energy (keV) of a virtual monochromatic PCD-CT image is shown on the horizontal axis. For reference, the CNR of iodine on an EID CT image is also shown on the vertical axis. In modules that contained iodine concentrations of 10-, and 40 mgI/ml, we observed the highest CNR on PCD CT scans at an effective energy near 60 keV. At both iodine concentrations, the highest CNR on PCD CT scans was higher than on EID CT images. These data were unpublished our own data.

## Clinical potential of photon-counting detector CT

### Dose reduction

The radiation exposure sustained at clinical CT studies remains a concern [18]. Dose-reducing techniques, e.g., automatic dose modulation, iterative reconstruction, automatic exposure control, electrocardiography-triggered imaging, and the placement of image filters have been developed. At the same level of X-ray exposure, the image noise is lower on PCD CT than conventional EID CT scans because PCDs minimize electronic noise and apply optimal X-ray photon energy-weighting. Consequently, PCD CT yields images of better quality than EID CT at lower radiation dose settings. Symons et al. [19] reported that at lower radiation doses, Hounsfield unit (HU) stability was greater and reproducibility was better with PCD CT than EID CT for images of lung-, ground-glass-, and emphysema equivalent foams. This indicates that PCD CT may help to reduce radiation exposure at lung cancer screenings and that it maintains diagnostic quality.

A radiation dose reduction is especially important in patients undergoing multiple CT studies. In patients at potential risk for cardiovascular events, the coronary artery calcium (CAC) level including the Agatston score, which is based on the amount of CAC detected on CT scans, is determined iteratively. According to a phantom study of Mergen et al. [20], CAC scoring with PCD CT is accurate at various tube voltages, potentially offering a substantial radiation-dose reduction. van der Werf et al. [21], who also performed a phantom study using medium- and high-density CAC, reported that PCD CT yielded reproducible Agatston scores at an up to 67% reduction in the radiation dose.

On CT scans of obese patients, the high image noise due to X-ray photon starvation renders the acquisition of high-quality images difficult. The quality degradation of their abdominal scans is particularly problematic because the high image noise may obscure subtle low-contrast lesions in parenchymal abdominal organ [22, 23]. Since PCDs are robust to photon starvation due to their improved noise behavior [24], they are expected to improve the image quality in large-bodied and obese patients. Decker et al. [25] compared the image quality of low-dose abdominal CT scans performed with PCD CT and EID CT. They found that on EID CT scans the body mass index (BMI) affects the noise and the signal-to-noise ratio more strongly than on PCD CT scans. Their findings suggest that low-dose PCD CT is a reliable option in patients with a high BMI.

### Improvement of spatial resolution

**Fig. 12:**
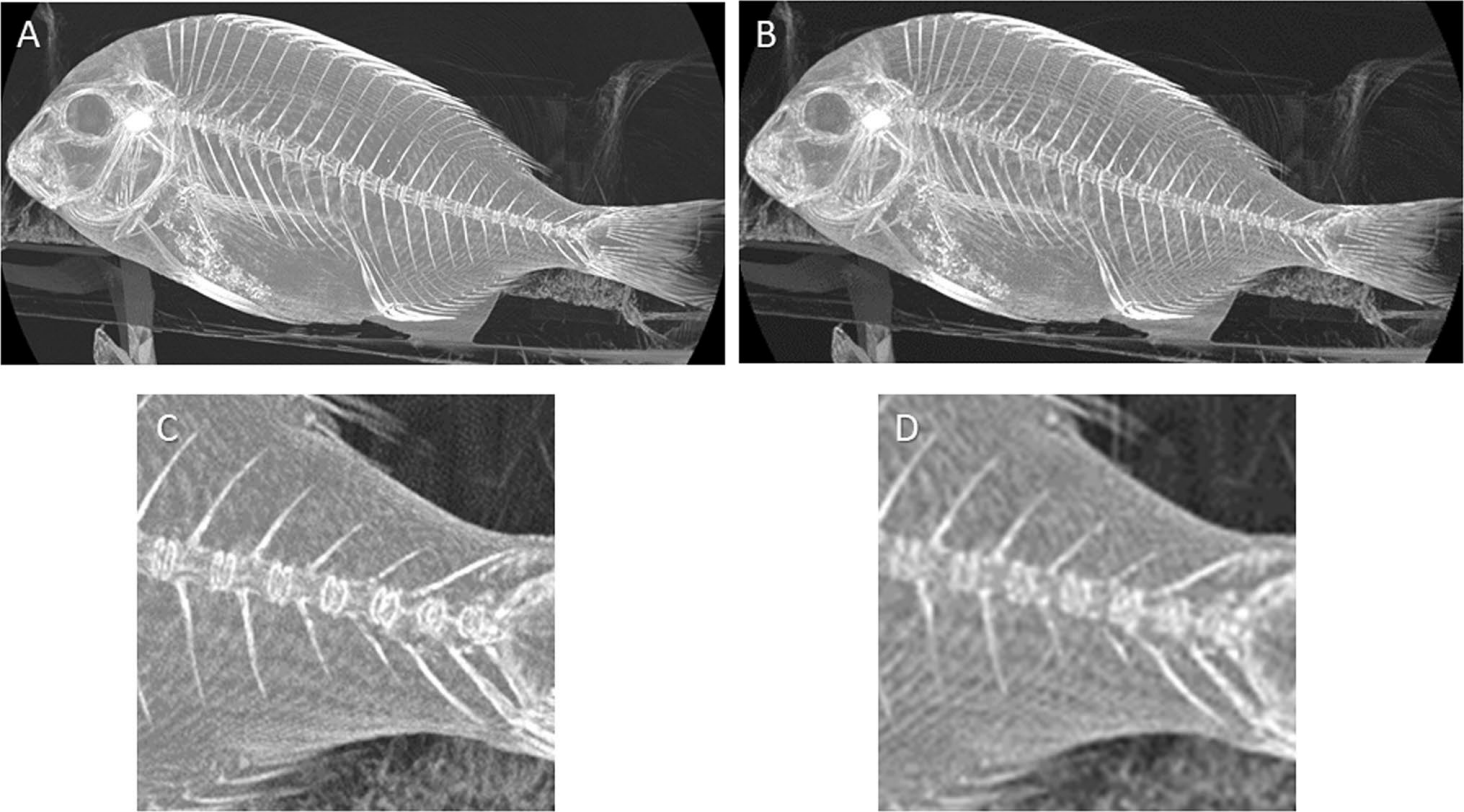
Maximum intensity projection images of fish (unpublished our own data). **A** PCD CT image in high resolution mode (matrix: 1024 × 1024, slab thickness: 45.75 mm). **B** PCD CT image with low resolution equivalent to conventional EID CT (matrix: 512 × 512, slab thickness: 45.75 mm). **C** Magnification of (**A**). **D** Magnification of (**B**). The overall structure is sharply delineated in (**A** and **C**) compared with (**B** and **D**)

Since the pixel electrode size is smaller on PCD CT scanners than the EID CT detector element, PCD CT can yield images with high spatial resolution (Fig. 12). Increased spatial resolution results in a decrease of partial volume effects and blooming artefacts, which are especially important for high-contrast materials, such as iodinated contrast, bone, and calcium. A higher spatial resolution may be useful for evaluating small structures and for identifying coronary artery calcium and coronary artery plaques, diagnosing lung lesions and temporal bone lesions.

#### Cardiac CT

The accurate and precise assessment of CAC is of clinical importance because it is strongly associated with future cardiovascular events.

For CAC detection, PCD CT is superior to conventional EID CT because PCD CT can more accurately measure the physical CAC volumes [26, 27]. The accuracy of CAC quantification is affected by blooming artifacts around CAC; these increase inter- and intra-scan variability. A dynamic anthropomorphic phantom study [28] revealed that PCD CT, but not conventional EID CT, provided reproducible Agatston scores at a heart rate of < 60 bpm regardless of the CAC density.

Coronary CT angiography (CCTA) is recommended for the assessment of many cardiovascular diseases and for the evaluation of coronary artery disease. The spatial resolution and soft-tissue contrast are limited on CCTA images acquired with conventional EID CT; this impairs their diagnostic performance with respect to small arteries (< 2 mm) and high-contrast (e.g., stents, calcification) and low-contrast (e.g., noncalcified plaques) studies. In addition, it carries the risks of relatively high X-ray exposure. Si-Mohamed et al. [29] compared the quality of CCTA images obtained with PCD CT and EID CT in humans. They reported that the image quality and diagnostic confidence were higher with PCD CT than EID CT. PCD CT also outperformed EID CT for the detection of lipid-rich atherosclerotic plaques [30]. At matched protocol settings and identical image reconstruction parameters, the in-stent lumen delineation of coronary artery stents was better on PCD CT- than conventional EID CT scans [31]. A comparison of specialized high-resolution PCD CT, EID CT, and standard PCD CT showed that despite an increase in the noise, the visualization of coronary plaques was improved and stent artifacts were decreased on PCD CT scans [32, 33].

#### Chest CT

By correlation with histopathologic studies and functional clinical testing, high-resolution CT (HRCT) has advanced the understanding of interstitial lung disease [34]. A detailed morphological evaluation of pulmonary nodules observed on HRCT images is essential because their size, shape, and growth are related to the likelihood of malignancy [35]. As the spatial resolution is higher on PCD CT- than EID CT images, it may improve the ability to diagnose lung diseases. In fact, visualization of higher-order bronchi, bronchial walls and pulmonary nodule was improved with PCD CT compared with EID CT [35, 36].

#### Bone CT

Temporal bone structures of clinical interest, such as the ossicles, facial nerve, and labyrinth, are submillimeter and require high-spatial-resolution imaging. Zhou et al. [37] reported that PCD CT in ultra-high-resolution mode was superior to EID CT in ultra-high-resolution mode for the delineation of the fine anatomy of the temporal bones.

Because of limited spatial resolution wrist trauma evaluation remains a challenging task for radiologists with the current conventional EID CT. Especially articular affliction and subtle trabecular fractures of the distal forearm and carpal bones can be difficult to evaluate [38]. A study of cadaveric wrist images showed that PCD CT may allow for a considerable radiation dose reduction and that the visualization of the fine anatomy was much better on PCD CT- than EID CT scans [39].

### Reduction of artifacts

As photons pass through the scanned object, low-energy- rather than high-energy photons are preferentially attenuated. Since polyenergetic beams are used in CT, this cause the effective photon energy to be shifted toward the higher end of the spectrum. This is known as beam hardening. Because PCD CT sorts individual photons based on their energy level, an energy bin image can be reconstructed using only higher-energy photons. The high-energy-bin image is more immune to beam-hardening effects on PCD CT- than EID CT- or low-energy PCD CT images. On the high-energy-bin image, calcium blooming, typically observed around the interface between the cranial bones and the brain, is reduced [8] and on virtual monochromatic images beam-hardening artifacts are eliminated [40].

### Virtual monochromatic image

Similar to DECT, the inherent spectral information of PCD CT can be used to calculate virtual monochromatic images (VMI). On low keV level VMI derived from DECT with EID CT, the iodine contrast is improved because the maximum iodine attenuation is close to the K edge of iodine (33.2 keV). However, the clinical application of DECT with EID CT is limited because the image noise is increased [41]. As the electronic image noise on PCD CT scans is lower than on EID CT images, PCD CT may improve the image quality of low keV level VMI. Zhou et al. [42] studied abdominal phantoms of three different sizes featuring iodine inserts of different concentrations to compare the image quality of VMI derived from PCD CT at different energy levels with conventional polychromatic EID CT images. They found that compared to the EID CT images, the 50 keV VMIs with PCD CT yielded a significantly higher objective image quality across all phantom sizes. Euler et al. [43] reported that the CNR was significantly higher on aortic PCD CT angiograms obtained with 40 keV and 45 keV VMI than on 80 kV EID CT scans and that the overall image quality was not degraded. This indicates that PCD CT may improve the image quality of lower VMI compared to EID-based DECT.

Multi-energy CT imaging of large patients with conventional dual energy EID CT is challenging due to photon starvation-induced image artifacts, especially in lower tube potential (80–100 kV) images. According to Tao et al. [44], on phantoms emulating obese patients, dual source PCD CT out-performed dual source EID CT with respect to iodine- and water-based material decomposition because it reduced image artifacts and improved iodine quantification.

### Virtual non-contrast image

Virtual non-contrast (VNC) imaging is one of the most investigated applications of dual energy EID CT because it may help to reduce the radiation dose by replacing true non-enhanced CT (TNCT) studies. However, at present, VNC imaging based on dual energy EID CT cannot replace TNCT because its iodine subtraction is inhomogeneous, the attenuation of calcifications and metallic clips is reduced, and the attenuation measured on VNC- and TNCT images is substantially different [45]. Sartoretti et al. [46] assessed the quality of hepatic VNC images in phantom- and patient studies. They reported that PCD CT allows for the reconstruction of VNC images of the liver both in a phantom and in patients with accurate attenuation values, being independent of dose, attenuation of base material, and liver iodine content compared with dual energy EID CT [46]. However, Niehoff et al. [47] reported that difference between TNC and VNC derived from PCD CT for abdominal CT images was 10 HU or less in 40% and 15 HU or less in 72%. Therefore, special caution should be excited when using VNC images in routine clinical practice even with PCD CT since there may be a small but significant difference in CT values between VNC- and TNCT images.

### Material decomposition

Iodine is the most common contrast agent used in clinical CT exams and many diagnoses rely on the enhancement of iodine signal on CT images. Ideally, as PCD CT yields perfect spectral separation, accurate iodine quantification can be expected. However, the performance of PCD CT in terms of iodine quantification was lower than expected due to charge-sharing and K-escape [48].

Sn filters filter out unnecessary photons. The addition of an Sn filter for DECT improves energy separation because the filter hardens the higher kVp beam (typically 140 kVp or 150 kVp) by selectively absorbing low energy photons. Thereby the filter improves the energy separation between low and high kVp images [49]. As EID-based DECT with an Sn filter currently yielded better performance than single source PCD CT without the filter [50], dual-source PCD CT with an Sn filter may result in better iodine quantification than single source PCD CT and EID-based DECT [51].

### K-edge imaging

**Fig. 13:**
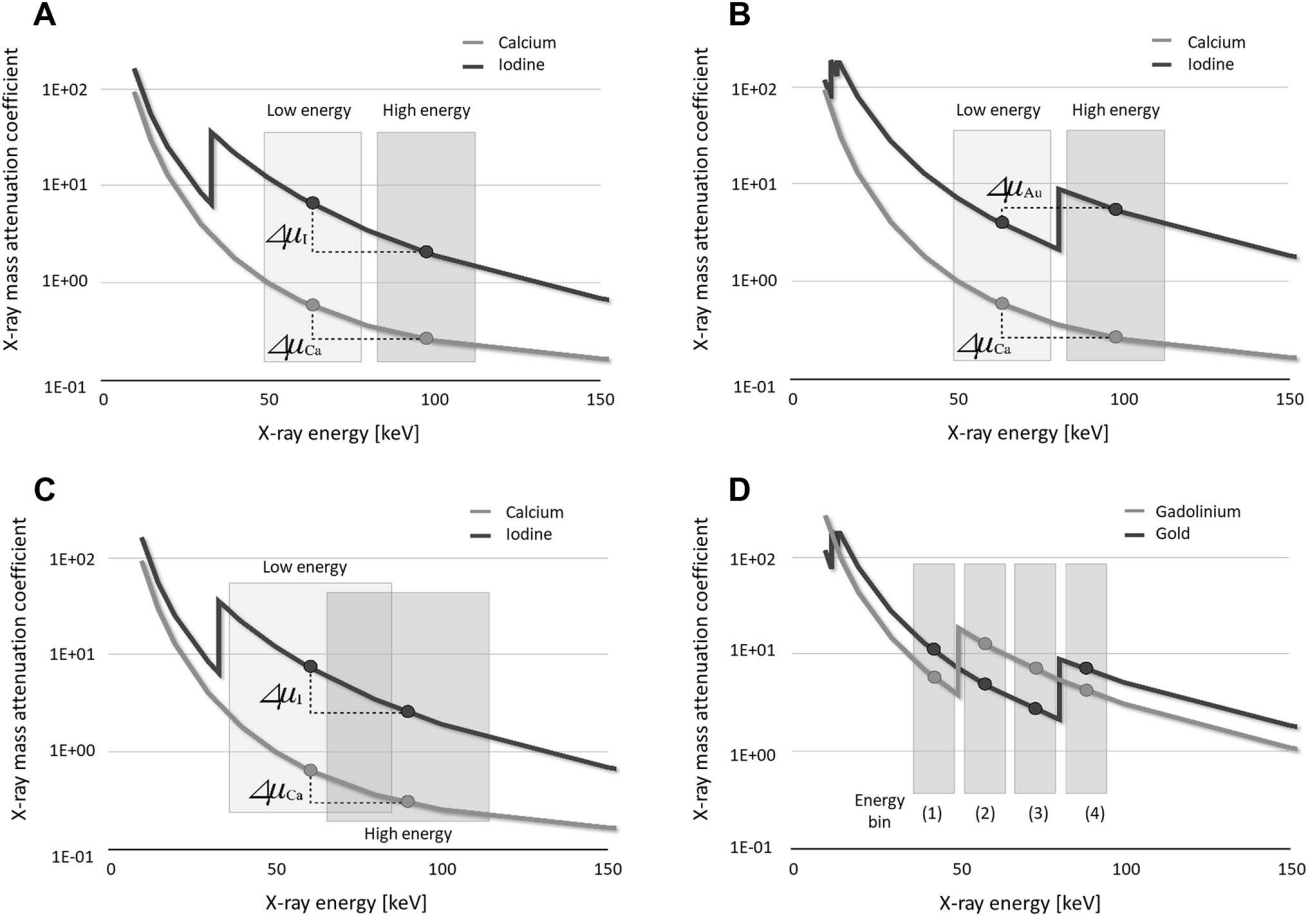
Principle of K-edge imaging at PCD CT. Material decomposition is performed based on the difference in the attenuation coefficients obtained at different energies (**A**). At K-edge imaging different energies are set on higher and lower level than K-edge of the target material (**B**). As 70–100 kVp and 135–150 kVp are routinely set for EID-based DECT scanning, these energies do not necessarily involve the K-edge of the materials which are used clinically such as iodine. In addition, the two energy spectra used have an overlap (**C**). At PCD CT scanning its energy can be classified into several energy bins and less overlap of different energy spectra (**D**)

As material decomposition is performed based on the difference in the attenuation coefficients obtained at different energies (Fig. 13A), the larger the difference in the attenuation coefficients, the more accurate decomposition can be expected. K-edge describes a sudden increase in the attenuation coefficient of photons occurring at a photon energy just above the binding energy of the K shell electron of the atoms interacting with the photons. Thus, for accurate material decomposition different energies should be set on higher and lower level than K-edge of the target material where the attenuation coefficients increase suddenly (Fig. 13B). This is called K-edge imaging. However, as two specific energy territories (e.g., 70 kVp and 135 kVp) are routinely set for EID-based DECT scanning [52], these energy territories cannot necessarily properly involve the K-edge of the materials which are clinically used such as iodine. In addition, as tube voltage set in CT only reflect the peak energy of the X-ray photons, spectral overlap definitely occurs at EID-based DECT (Fig. 13C) [53]. Thus, although differentiation of materials with EID-based DECT works especially well when the two energy spectra used have a minor overlap, the acquisition of stable and accurate material decomposition presents a challenge when the number of materials in a mixture is more than two and one or more of the components have distinctive K-edges [54, 55]. This challenge may be overcome with PCD CT because its energy can be classified into several energy bins and less overlap of different energy spectra (Fig. 13D), allowing K-edge imaging for various materials. Combining material decomposition and the third-basis function for an atom in the contrast agent of interest (e.g., iodine, gadolinium) makes it possible to quantify the spatial distribution of the contrast agents on a pixel basis [56].

Elements with high atomic numbers (e.g., iodine, gadolinium, gold, bismuth) can be identified by K-edge imaging [57, 58]. Using high atomic number elements such as gold, nano particles (specific to certain cells or enzymes) are labeled and detected by K-edge imaging at PCD CT for molecular imaging. Molecular CT imaging may be useful for diagnosing early-stage cancers because it detects and quantifies small tumors [59], and it may also help in the evaluation of atherosclerotic plaques [6, 60].

**Fig. 14:**
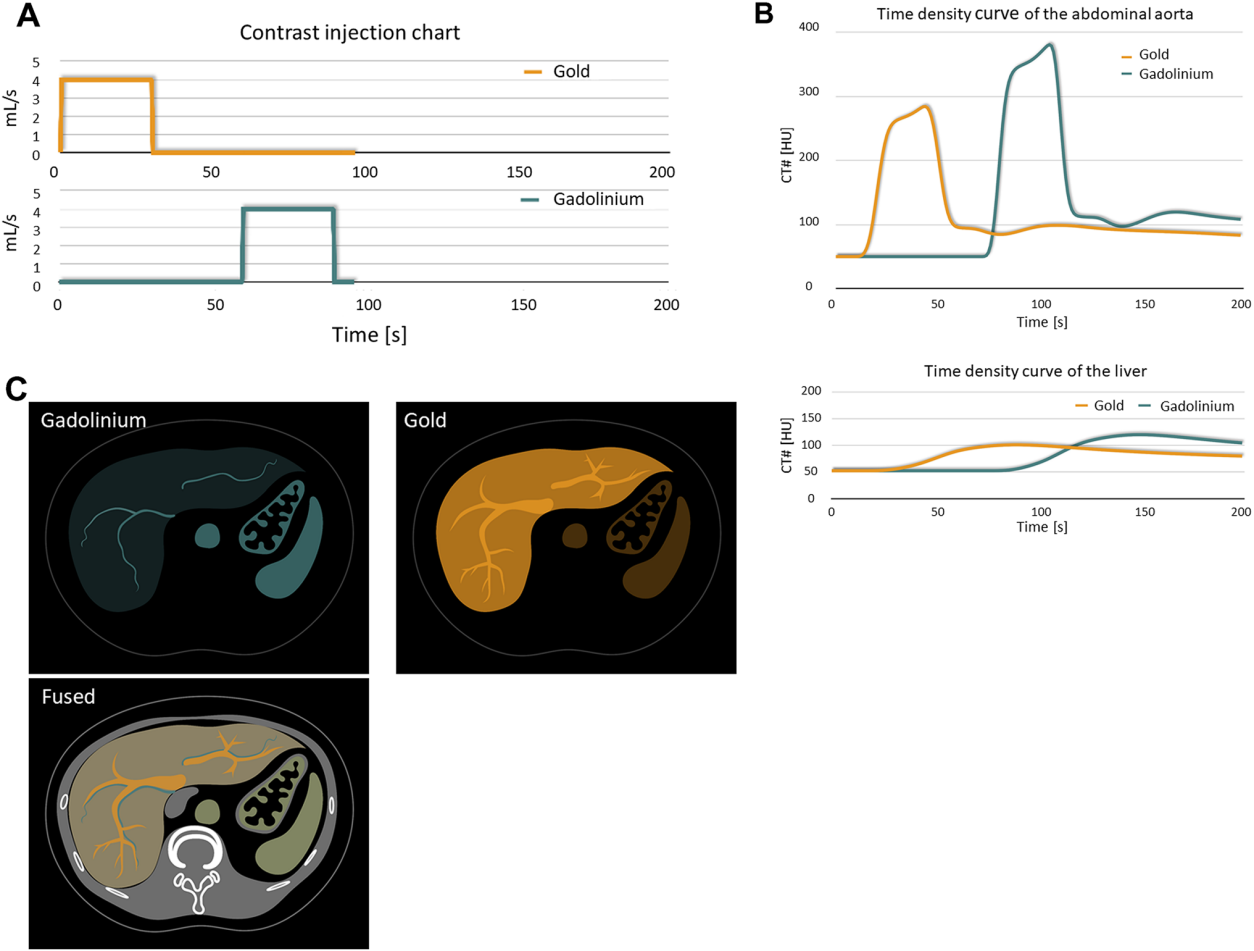
Simulated single-scan dual-contrast biphasic liver imaging using K-edge imaging. Scanning protocol to simultaneously capture maximum enhancement of gadolinium during the late arterial phase and of gold during the portal venous phase. **A** contrast material injection timing chart, **B** simulated time intensity curve of the abdominal aorta and liver. **C** Arteries containing gadolinium-based contrast agent can be visualized on gadolinium maps. Portal veins and liver parenchyma containing gold-based contrast agent can be visualized on gold maps

The administration of different contrast agents at different times makes it possible to obtain multiple-phase images at a single time point (Fig. 14). Ren et al. [61] used a dual-contrast (iodine and gadolinium) injection protocol to study simultaneous biphasic porcine liver images acquired during a single PCD CT acquisition. The hepatic arteries (containing iodine) and the hepatic veins (containing gadolinium) were clearly visualized and delineated. This technique was also useful for detecting endoleaks on a single scan performed after endovascular aortic repair [62].

Multiple-phase imaging at a single time point using different contrast agents may obviate the need for multi-phase CT scans, thereby reducing the delivered radiation dose [62, 63]. However, a much higher amount of gadolinium (about 10 times the dose of clinical MRI) is needed for PCD CT imaging and other contrast agents such as gold and bismuth are not approved for human examinations. Therefore, it is unclear at this time whether K-edge imaging can be performed in clinical PCD CT examinations.

### Current issue of the PCD CT

Although the image quality of PCD CT scans is superior to that of EID CT currently, further improvement may be possible as follows. While dual-contrast imaging is currently possible, good images cannot necessarily be obtained with usual radiation dose due to a significant noise increase through material decomposition process [64, 65]. Iterative reconstruction- [66] and deep convolutional neural network-based [61] denoising techniques may be useful and future improvements of the image quality can be expected.
